# Immunotherapy in Nonendemic Nasopharyngeal Carcinoma: Real-World Data from Two Nonendemic Regions

**DOI:** 10.3390/cells11010032

**Published:** 2021-12-23

**Authors:** Panagiota Economopoulou, Anastasios Pantazopoulos, Aris Spathis, Ioannis Kotsantis, Anastasios Kyriazoglou, George Kavourakis, Roubini Zakopoulou, Ioannis Chatzidakis, Maria Anastasiou, Maria Prevezanou, Carlo Resteghini, Lisa Licitra, Cristiana Bergamini, Elena Colombo, Francesca Caspani, Nerina Denaro, Stefania Vecchio, Pierluigi Bonomo, Maria Cossu Rocca, Federica Bertolini, Daris Ferrari, Amanda Psyrri, Paolo Bossi

**Affiliations:** 1 Section of Medical Oncology, Second Department of Internal Medicine, Attikon University Hospital, National and Kapodistrian University of Athens, 12462 Athens, Greece; panagiota_oiko@hotmail.com (P.E.); anastasios.pantazopoulos@gmail.com (A.P.); ikotsantis@gmail.com (I.K.); tassoskyr@gmail.com (A.K.); georgekavourakis@yahoo.gr (G.K.); Rzakopoul@gmail.com (R.Z.); biovazelos@hotmail.com (I.C.); miriamanastasiou9@gmail.com (M.A.); mariaprevezanou@gmail.com (M.P.); 2 Second Department of Pathology, Attikon University Hospital, National and Kapodistrian University of Athens, 12462 Athens, Greece; arisspa@gmail.com; 3 Head and Neck Medical Oncology Unit, Fondazione IRCCS Istituto Nazionale Dei Tumori, Via Venezian 1, 20133 Milan, Italy; carlo.resteghini@istitutotumori.mi.it (C.R.); lisa.licitra@istitutotumori.mi.it (L.L.); cristiana.bergamini@istitutotumori.mi.it (C.B.); Elena.Colombo@istitutotumori.mi.it (E.C.); francesca.caspani@istitutotumori.mi.it (F.C.); 4 Department of Oncology and Hemato-Oncology, University of Milan, 20122 Milan, Italy; 5 Medical Oncology Santa Croce and Carle General Hospital Cuneo, 12100 Cuneo, Italy; nerinadenaro@gmail.com; 6 IRCCS Ospedale Policlinico San Martino, 16132 Genova, Italy; stefania.vecchio@hsanmartino.it; 7 Radiation Oncology, Azienda Ospedaliero-Universitaria Careggi, 50134 Florence, Italy; bonomopierlu@gmail.com; 8 Department of Medical Oncology, Urogenital and Head and Neck Tumors Medical Treatment, IEO, European Institute of Oncology IRCCS, 20141 Milan, Italy; maria.cossurocca@ieo.it; 9 Medical Oncology Unit, Department of Oncology and Hematology, University Hospital of Modena, 41125 Modena, Italy; bertolini.federica@policlinico.mo.it; 10 Medical Oncology Unit, San Paolo Hospital, 20142 Milan, Italy; daris.ferrari@asst-santipaolocarlo.it; 11 Medical Oncology, Department of Medical and Surgical Specialties, Radiological Sciences and Public Health, University of Brescia, ASST-Spedali Civili, 25123 Brescia, Italy; paolo.bossi@unibs.it

**Keywords:** immunotherapy, nasopharyngeal cancer, EBV DNA, nonendemic region

## Abstract

Background: nasopharyngeal carcinoma (NPC) is a complex disease entity that mainly predominates in endemic regions. Real-world data with immunotherapy from nonendemic regions are limited. Methods: we collected data from patients with recurrent/metastatic (R/M) NPC treated at a center in Greece and 8 centers in Italy. Between 2016 and 2021, 46 patients who were treated with at least one cycle of immune checkpoint inhibitors (ICI) were identified. Herein, we present our results and a review of the literature. Results: assessment of response was available in 42 patients. Overall, 11 patients responded to immunotherapy (Overall Response Rate-ORR 26.2%). Three patients had complete response (CR), and 8 patients had partial response (PR). Disease control rate (DCR) was 61.9%. Median Progression Free Survival (PFS) was 5.6 months and median Overall Survival (OS) was 19.1 months. Responders to ICI improved PFS and OS as compared to that of nonresponders. A lower probability of responding to ICI was shown in patients with more than three metastatic sites (*p* = 0.073), metastatic disease at initial diagnosis, (*p* = 0.039) or EBV DNA positive before ICI initiation, (*p* = 0.074). Decline in EBV DNA levels was found to be statistically significant associated with best response to ICI (*p* = 0.049). Safety was manageable. Conclusions: among 46 patients with R/M NPC treated with immunotherapy in two nonendemic regions, ORR was 26.2% and durable responses were observed. Low disease burden could serve as a biomarker for response to ICI.

## 1. Introduction

Due to its distinct and multifaceted etiology, cancer of the nasopharynx (NPC) exhibits a specific geographic distribution. Thus, it is an endemic malignancy in Asian populations, such as Southern China and Southeast Asia and very rare in the United States (US) and Western Europe [1]. In endemic areas, the development of NPC was linked to a combination of Epstein–Barr virus (EBV) infection, lifestyle factors such as tobacco consumption and various genetic changes in crucial genes/pathways, such as *CDKN2A*, *CCND1* and *TP53* [2,3]. In nonendemic areas, such as Europe and the United States, sporadic NPC is mainly associated with smoking and alcohol use, similar to other squamous carcinomas of the head and neck (SCCHN), even if a significant proportion is linked to EBV, too [2].

Advances in radiotherapy techniques and combination chemotherapy strategies have boosted 5-year survival rates to almost 80% depending on disease stage [4]. Following the wide incorporation of intensity-modulated radiation therapy (IMRT) and the larger use of chemotherapy in the treatment algorithm of early/locally advanced NPC, the cumulative rate of nasopharynx and cervical lymph node recurrence decreased to 10–20% at 5 years [5]. Regarding recurrent/metastatic (R/M) disease treatment options are limited. In Europe, cytotoxic cisplatin-based chemotherapy is currently the mainstay of treatment in the first-line setting, leading to high response rates [6]; in patients with chemotherapy-sensitive de novo metastatic disease and a response to first-line chemotherapy, a survival benefit was shown from consolidation radiotherapy in a randomized trial [7].

More recently, two parallel clinical trials conducted in China showed that two novel monoclonal antibodies against immune checkpoint Programmed Cell Death-1 (PD-1), toripalimab and camrelizumab, extend overall survival (OS) in combination with cisplatin-based chemotherapy in patients with previously untreated R/M NPC [8,9]. Following these results, immunotherapy is expected to be incorporated in the treatment algorithm of advanced disease soon. On the other hand, pembrolizumab and nivolumab, two agents that have received Food and Drug Administration (FDA) approval for SCCHN in the R/M setting, have demonstrated promising antitumor activity in phase Ib/II trials in patients with previously-treated NPC [10,11]; however, pembrolizumab failed to improve OS compared to investigator’s choice chemotherapy in the recently presented phase III Keynote-122 clinical trial in platinum-pretreated R/M NPC [12].

Importantly, immunotherapy studies in NPC were predominately conducted in endemic areas and the majority of patients included are of Asian origin [8,9,10,11]. Nevertheless, there is limited data regarding the use of ICIs in patients with advanced NPC in nonendemic areas. In this report, we sought to depict our experience regarding the use of immune checkpoint inhibitors (ICIs) in patients with advanced NPC in the nonendemic areas of Greece and Italy.

## 2. Materials and Methods

### 2.1. Patients

We reviewed the institutional databases of one reference center in Greece (Attikon University Hospital, Athens, Greece) and eight centers in Italy (Fondazione IRCCS Istituto Nazionale dei Tumori (INT), Milan, Italy, IEO-European Institute of Oncology IRCCS Milan, Italy, San Paolo Hospital, Milan, Italy, S Croce and Carle Teaching Hospital, Cuneo, Italy, IRCCS San Martino-IST National Cancer Institute and University of Genova, Genova, Italy, Azienda Ospedaliero-Universitaria Careggi, Florence, Italy, Modena University Hospital, Modena, Italy, and ASST-Hospital Spedali Civili, Brescia, Italy,) to identify patients with R/M NPC that have received at least one dose of ICI (nivolumab or pembrolizumab) between August 2016 and May 2021. Clinical details such as demographics (age, sex, tobacco, alcohol consumption, and comorbidities), disease characteristics (histology according to WHO classification, date of first diagnosis, TNM stage, site of metastasis), treatment information (type of ICI, line of therapy, number of cycles administered, type of previous and subsequent therapies, response to ICI, immune-related adverse events-irAEs), biochemical parameters (EBV DNA copy number quantification at diagnosis and change post-treatment) and patient survival were obtained.

The present study was approved by the Medical Ethical Committee of Attikon University hospital (Athens, Greece) and of Fondazione IRCCS Istituto Nazionale dei Tumori, (Milan, Italy) as part of the NPC Portal project (Approval number 204/17). The study complies with the principles laid down in the Declaration of Helsinki.

Stage was calculated according to the Tumor Node Metastasis (TNM) classification of the American Joint Committee for Cancer staging 8th edition [13]. Charlson comorbidity index was used for assessment of comorbidities [14]. When available, EBV DNA copy number was quantified by real-polymerase chain reaction (PCR) in blood specimens. Response to ICI was assessed using computed tomography (CT) or magnetic resonance imaging (MRI) of the head and neck and CT scan of the thorax and abdomen, which were done every three months or as clinically needed. Positron Emission Tomography (PET)/CT was performed when clinically indicated. Response to ICI was evaluated using the Response Evaluation Criteria in Solid Tumors 1.1.IrAEs were defined based on specific criteria of previous immunotherapy studies [15]. Grade of irAEs was defined according to Common Terminology Criteria for Adverse Events version 5.0.

### 2.2. Statistical Analysis

Statistical analysis was performed using SPSS 25. Categorical data were compared using fisher’s exact test and correlations were examined using nonparametric tests (Spearman). Survival analysis was performed using Kaplan Meyer, and multiparametric cox survival analysis was used to identify independent prognostic factors. Progression-free survival (PFS) was estimated from the start date of treatment with ICI to the date of disease progression or death from other causes or censored at the last date of follow-up. Overall survival (OS) was estimated from the start date of treatment with ICI to the date of death or censored at the last date of follow-up.

## 3. Results

### 3.1. Patient Characteristics

We identified 46 patients with R/M NPC who were treated with ICI during their disease management. Baseline characteristics of patients are shown in Table 1. Median age was 56.3 years (range 32 to 77 years old); ten patients were female (21.7%), and the rest were male (36 patients, 78.3%). Seventeen patients (37%) had recurrent and 29 (63%) had metastatic disease. Eight patients (17.4%) had metastatic disease at initial diagnosis. At the time of relapse, 9 patients (19.5%) experienced local relapse only at primary site, 6 (13%) only at regional lymph nodes, 9 patients (19.5%) had local relapse at both primary site and regional lymph nodes, 15 (32.6%) had metastatic disease only and 3 (6.5%) had both metastases and local relapse. Among patients with metastatic disease, 10 (21.7%) patients had pulmonary metastases, 16 (34.8%) patients had osseous metastases and 11(23.9%) had hepatic metastases.

### 3.2. Treatment, Response and Toxicity

The majority of patients received nivolumab (42 patients, 91.3%). Four patients received pembrolizumab (8.7%). Nivolumab was administered as initial treatment for R/M disease in 6 patients (13%), as second line treatment in 21 patients (45.7%), as third line treatment in 5 patients (10.9%) and as fourth line and beyond in 10 patients (21.7%). Pembrolizumab was administered as first-line treatment in 2 patients (4.3%), as second line treatment in one patient (2.1%) and as fourth line treatment in one patient (2.1%). The median number of cycles delivered was 14 (range, 1–58). All patients who received nivolumab and pembrolizumab as initial treatment for R/M NPC had platinum-resistant disease (progression < 6 months from the last platinum dose) following concurrent chemotherapy and radiation for locally advanced NPC. When nivolumab was administered as second-line therapy and beyond, first-line treatment consisted of platinum-based chemotherapy in the majority of patients (32 patients, 69.6%).

Forty-two patients were eligible for assessment of response at the time of the analysis. Among them, 11 patients (26.2%) responded; three patients had complete response (CR) (7.1%) and 8 (19.1%) had partial response (PR) to immunotherapy. Fifteen patients (35.7%) had stable disease (SD), and the remaining 16 patients (38.1%) had primary resistance to nivolumab-progressive disease (PD). Disease control rate, defined as the rate of patients with CR, PR and SD was 61.9%. Importantly, the majority of responders (8 patients, 72.7% of responders) had durable responses of more than a year and five patients (45.4% of responders) still remain on treatment (range of cycles administered 9–58).

Pre- and postimmunotherapy plasma EBV DNA copy number was available in 31 patients. Following IO treatment, EBV change was detected in 12 patients (37.5%); decline in EBV copy number was observed in 6 patients and increase in 6 patients. Patients with positive plasma EBV DNA levels before IO initiation had a trend for higher probability to experience PD (*p* = 0.074). Patients who had increase in EBV DNA levels were more likely to experience PD (χ^2^, *p* = 0.030). In addition, EBV DNA decline was statistically significant correlated with best response to ICI (CR, PR) (χ^2^, *p* = 0.047).

There was no association of response to IO with the number of prior lines of therapy. Patients with less than 3 metastatic sites at the time of ICI initiation had a trend for higher probability of responding to immunotherapy (*p* = 0.073) and were statistically significant less likely to experience PD (*p* = 0.031) and PD in less than 6 months (*p* = 0.053). Patients with bone metastases were more likely to experience PD to immunotherapy (*p* = 0.054). In addition, patients with metastatic disease at initial presentation of NPC were more likely to have PD in less than 6 months’ time (*p* = 0.039). No other demographic factors (sex, tobacco, alcohol consumption, comorbidities) or disease characteristics (stage, history of concurrent CRT use, type of relapse) were associated with response to ICI.

Fourteen patients had an immunotherapy-related adverse event. In two patients, the occurrence of the irAE (grade 3 hepatitis and grade 3 myasthenia gravis) led to permanent ICI discontinuation. Another two patients had grade 3 abnormalities in laboratory values (AST and lipase increase), which resulted in temporary ICI interruption. The remaining three patients had grade 1–2 irAEs (diarrhea, fatigue, thyroiditis, pneumonitis, and type II diabetes).

### 3.3. Association with Survival

At the time of writing, 20 patients had died. Median overall survival (OS) was 19.1 months (95% CI: 11.37−26.76+). Median progression-free survival (PFS) was 5.6 months (95% CI: 0.56−10.74+).

**Figure 1:**
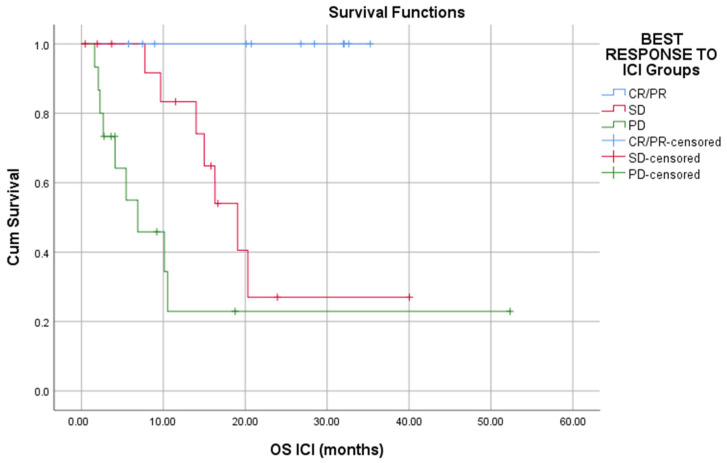
Responders to ICI have better overall survival as compared to that of nonresponders (*p* < 0.01).

**Figure 2:**
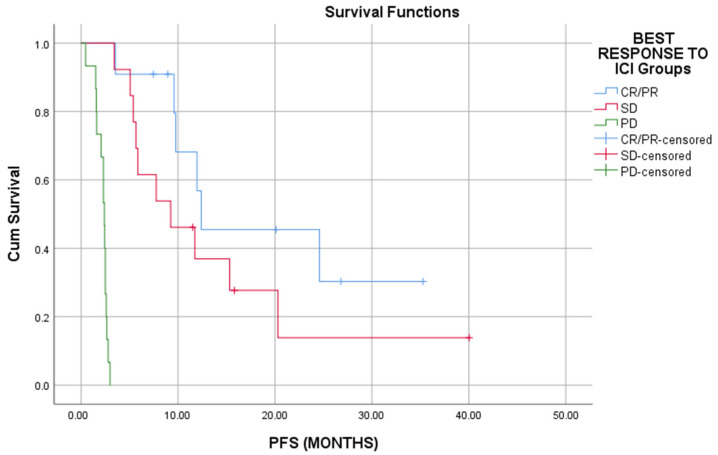
Responders to ICI have better progression free survival as compared to that of nonresponders (*p* < 0.01).

Responders to ICI had a statistically significant prolonged OS and PFS than patients who had SD or PD (Long rank, *p* < 0.01) (Figure 1 and Figure 2).

**Figure 3:**
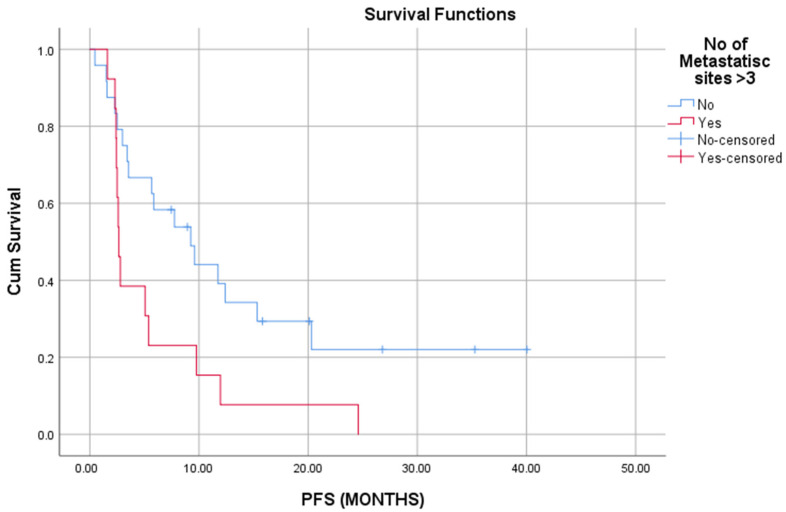
Presence of more than three metastatic sites is associated with worse PFS (*p* = 0.023).

Similarly, patients who experienced PD had worse OS (long rank, *p* = 0.015); patients that had PD in less than 6 months had also shorter OS and PFS (long rank, *p* < 0.05). The presence of more than 3 metastatic sites at IO initiation was associated with reduced PFS (long rank, *p* = 0.23) (Figure 3).

No other demographics or disease features correlated with survival. Multivariate Cox proportional hazard models did not reveal any independent prognostic factors for PFS or OS (Table 2).

## 4. Discussion

Owing to established geographic variation, endemic NPC dominates in Southern China, whereas sporadic cases occur more frequently in Southeast Asia, North Africa and the Middle East and rarely in the US and Western Europe [1]. Based on WHO classification, NPC is categorized in three histological subtypes: nonkeratinizing squamous cell, keratinizing squamous cell and basaloid carcinoma [16]. In endemic high-incidence areas, non-keratinizing carcinoma (either differentiated or undifferentiated) is the predominant histological subtype, which is correlated with EBV infection and is characterized by improved survival. On the contrary, in nonendemic areas, keratinizing squamous cell carcinoma comprises the majority of cases, might be etiologically linked to more traditional risk factors such as tobacco consumption and is associated with markedly less favorable survival [17,18].

Due to a potentially more achievable patient accrual, the majority of large, randomized studies of NPC originate from endemic areas. This unequal patient distribution complicates the compilation of data from patients in nonendemic areas and despite concordance in national clinical practice guidelines, the question whether results from Asian trials should be applicable to low-incidence regions still remains. In this report, we portray the experience regarding the use of ICI in patients with R/M NPC from two nonendemic areas, one head and neck cancer reference center in Greece, and eight centers in Italy. Among 42 patients with evaluable response to ICI, we report an overall response rate (ORR) of 26.2% and a DCR of 61.9%. Among responders, three patients had CR and 8 patients had PR. Most importantly, among these patients, eight had durable responses of more than a year and five of them continue on immunotherapy treatment (nivolumab). Of note, two patients who continue on ICI were heavily pretreated. We found that response to ICI statistically significant correlated with OS and PFS (*p* = 0.01)

We demonstrated a negative association of aggressive disease features (more than 3 metastatic sites, metastatic disease at initial diagnosis, and positive pretreatment plasma EBV DNA) with response to ICI. This was not observed in phase II study of nivolumab. However, comparable results were reported in metastatic melanoma, where patients with high disease burden (high number of metastatic sites, elevated LDH) were shown to have lower response rates to immunotherapy [19,20]. This finding could serve as a potential biomarker of ICI monotherapy preferably in patients with low disease burden. Nevertheless, patients with high disease burden might confer improved responses with combination of chemotherapy and immunotherapy.

Importantly, we found a correlation of best response to ICI (CR/PR) with decline in EBV DNA levels postimmunotherapy (*p* = 0.047). Negative pretreatment EBV DNA were shown to be a prognostic biomarker for improved PFS and OS in nonendemic areas [21]. In addition, EBV DNA change was found to correlate with response to pembrolizumab in eight patients included in the phase Ib Keynote-028 study [22]. Indeed, an increase in plasma EBV DNA may be an early surrogate marker of PD to immunotherapy, as it was shown with chemotherapy in the same setting.

The efficacy of nivolumab in R/M NPC was evaluated in a phase II international multicenter study that included 44 participants [11]. The majority of patients that took part in the study were Asian (82.2%), whereas Caucasian patients comprised a small percentage of the sample size (4 patients, 8.9%). Similar to our cohort, ORR was 20.5% (7 patients had PR) and DCR was 54%. Of note, three patients had SD which lasted more than one year [11].

Several additional studies have tested the antitumor activity of anti-PD-1 antibodies in R/M NPC. In the phase Ib study Keynote-028, 27 patients with PD-L1 positive R/M NPC received pembrolizumab; the majority of patients had been treated with prior therapies for advanced disease (range: 1–5 lines of treatment), whereas 2 patients received pembrolizumab as first line treatment [10]. This study demonstrated an ORR of 25.9% and a clinical benefit rate of 77.8%. In our cohort, four patients received pembrolizumab; however, PD-L1 was available only in one patient (CPS = 100). Among these patients, evaluation of response was not available in one, whereas one patient had PD and two patients had long-lasting disease control (SD for more than 10 months). In Keynote-028, approximately one third of patients experienced durable disease control with pembrolizumab [10]. Of note, a larger proportion of patients from nonendemic regions was included in this study (37%). However, the confirmatory phase III Keynote-122 clinical trial, which sought to evaluate the efficacy of pembrolizumab as compared to investigator’s choice chemotherapy in 253 patients with platinum-pretreated advanced NPC, failed to show any superiority of pembrolizumab over chemotherapy neither in the intention to treat population nor in the PD-L1 CPS ≥ 1 group (median OS 17.2 months vs. 15.3 months, *p* = 0.22) [12]. Of note, this study included only patients with EBV-positive disease. Nevertheless, ORR to ICI (21.4%) and median OS (17.2 months) was similar to our cohort.

Spartalizumab, an anti-PD-1 antibody, was compared to investigator’s choice chemotherapy in a randomized phase II study in patients with platinum-refractory NPC [23]. This study included 122 patients from endemic areas, among who 76 received spartalizumab. The study did not meet its primary endpoint of PFS (1.8 months in the spartalizumab arm vs. 6.6 months in the chemotherapy arm (HR = 1.53, 95% CI 1.02; 2.27)). ORR was 18.4% in the spartalizumab arm. Of note, 61% of spartalizumab responders had long lasting responses more than one year.

Anti-PD-1 antibodies camrelizumab and toripalimab were both evaluated in phase II studies in pretreated patients with R/M NPC in China. Camrelizumab produced an ORR of 28.2% with approximately half of patients reaching durable responses of one year or more [24]. In the phase II POLARIS-02 trial, toripalimab was tested in 190 patients with platinum refractory NPC, yielding an ORR of 20.5%, a median duration of response of 12.5 months and median OS of 17.1 months [25]. These promising results heralded the impressive findings of the incorporation of camrelizumab/toripalimab in the first line setting in combination with cisplatin-based chemotherapy. Captain-1st was a randomized, phase III, placebo-controlled study that evaluated the efficacy of camrelizumab in combination with chemotherapy in patients with untreated NPC in 28 hospitals in China [9]. It was found that the addition of camrelizumab prolonged the primary endpoint of PFS by 2.8 months (9.7 months vs. 6.9 months, HR = 0.54, *p* = 0.0002). Jupiter-02 was a placebo-controlled phase III trial that assessed the activity of toripalimab in combination with cisplatin-gemcitabine combination as first-line treatment in advanced NPC [8]. The combination of chemotherapy-toripalimab led to the improvement of ORR (77.4% vs. 66.4%) and PFS (11.7 vs. 8 months, HR = 0.52, *p* = 00003). The role of maintenance treatment in the survival advantage needs to be further evaluated, as it was allowed only in the experimental arms. Published studies evaluating ICIs in R/M NPC are shown in Table 3.

## 5. Conclusions

We present data from 46 patients with R/M NPC treated in Greece and Italy. In our cohort, ORR was 26.2% and DCR rate was 61.9%, similar the one reported in phase I/II studies of anti-PD-1 inhibitors in pretreated patients. Durable disease control has also been observed in responders. Importantly, aggressive disease features before ICI start and increase in EBV DNA levels post-ICI were shown to correlate with high probability of PD to immunotherapy. Published data on NPC are mainly based on studies originating from endemic regions and Caucasian patients are under-represented in those studies. Thus, little is known about differences in the efficacy of immunotherapy between Asian patients and patients from low-incidence regions. Indeed, several trials have shown differences in the activity of several drugs in Asian vs. non-Asian populations, such as S-1 in gastric cancer [26]. Due to difficulties in patient accrual in low-incidence regions, the conduction of large, randomized trials in non-endemic regions might not be feasible. Therefore, retrospective studies with larger number of patients might provide important information about treatment efficacy and prognosis in patients from low prevalence geographic regions. Identifying the patients who could obtain the highest benefit from ICI is the logical next step in research, by means of evaluating the genomic characteristics of patients achieving the long-term survival.

## Figures and Tables

**Table 1:** Baseline patient characteristics. Abbrev. EBV = Ebstein–Barr Virus, N/A: Not Available, TNM = Tumor Node Metastasis, WHO = World Health Organization.

Characteristic		N	%
Age	<65 years	35	76.1
	≥65 years	11	23.9
Sex	Male	36	78.3
	Female	10	21.7
History of tobacco use	Active smoker	14	30.4
	Former smoker	11	23.9
	Nonsmoker	21	45.7
History of alcohol consumption	No	41	89.1
	Moderate/Heavy	5	10.9
Charlson comorbidity index	≤2	16	34.8
	>2	22	47.8
	N/A	8	17.4
Site of metastasis	Lung	10	21.7
	Bone	16	34.8
	Liver	11	23.9
	Other	6	13
	None	17	36.9
Stage TNM	II–III	18	39.1
	IV	24	52.2
	N/A	4	8.7
Histology (WHO classification)	Nonkeratinizing squamous cell carcinoma	40	86.9
	Keratinizing squamous cell carcinoma	3	6.5
	Basaloid	1	2.2
Pretreatment plasma EBV DNA	N/A	2	4.4
	Positive	18	39.1
	Negative	13	28.3
	Unavailable	15	32.6

**Table 2:** Prognostic factors for overall survival. Abbrev. EBV = Epstein–Barr Virus, ICI = Immune Checkpoint Inhibitors.

Covariates	Univariate Anaylsis				Mutlivariate Analysis			
	HR	95.0% CI		*p*-Value	HR	95.0% CI		*p*-Value
Age (years)	1.011	0.969	1.056	0.612	1.002	0.910	1.103	0.970
Comorbidities	1.019	0.859	1.208	0.829	0.549	0.269	1.120	0.099
EBV DNA at first diagnosis	1.000	1.000	1.000	0.082	1.000	1.000	1.000	0.361
Pre-ICI EBV DNA	1.000	1.000	1.000	0.583	1.000	1.000	1.000	0.687
Line of therapy	1.026	0.735	1.431	0.881	1.569	0.680	3.618	0.291
No. of metastatic sites	1.002	0.894	1.124	0.966	0.919	0.582	1.451	0.716

**Table 3:** Anti-PD-1 studies in R/M NPC. Abbrev: ICI = Immune Checkpoint Inhibitor, NPC = Nasopharyngeal Carcinoma, NR = Not Reached, PFS = Progression Free Survival, R/M = Recurrent/Metastatic, ORR = Overall Response rate, OS = Overall Survival, Pts = patients.

Study/ICI	N of Pts (Total (Assigned to ICI))	Setting	Phase	Median PFS (Months)	Median OS (Months)	ORR %	Ref
NCI-9742/Nivolumab	45	Pretreated	II	2.8	17.1	20.5	[11]
Keynote-028/Pembrolizumab	27	Pretreated	Ib	6.5	16.1	25.9	[10]
Keynote-122/Pembrolizumab vs. chemotherapy	233 (117)	Platinum-refractory	III	4.1 vs. 5.5	17.2 vs. 18	21.4 vs. 23.3	[12]
PDR001/Spartalizumab vs. chemotherapy	122 (76)	Platinum-refractory	II	1.9 vs. 6.6	NR in either group	18.4 vs. 32.4	[23]
Captain-1st/Camrelizumab + chemotherapy vs. placebo + chemotherapy	263 (134)	1st line	III	9.7 vs. 6.9	NR vs. 22.6	87.3 vs. 80.6	[9]
Jupiter-02/Toparilimab + chemotherapy vs. placebo + chemotherapy	289 (146)	1st line	III	11.7 vs. 8	NR in either arm	77.4 vs. 66.4	[8]

## Data Availability

Data are contained within the article.
